# Managing the Cloud Continuum: Lessons Learnt from a Real Fog-to-Cloud Deployment

**DOI:** 10.3390/s21092974

**Published:** 2021-04-23

**Authors:** Xavi Masip-Bruin, Eva Marín-Tordera, Sergi Sánchez-López, Jordi Garcia, Admela Jukan, Ana Juan Ferrer, Anna Queralt, Antonio Salis, Andrea Bartoli, Matija Cankar, Cristovao Cordeiro, Jens Jensen, John Kennedy

**Affiliations:** 1 CRAAX Lab, Universitat Politècnica de Catalunya, 08800 Vilanova i la Geltrú, Spain; eva@ac.upc.edu (E.M.-T.); sergio@ac.upc.edu (S.S.-L.); jordig@ac.upc.edu (J.G.); 2 Communications Network Group, Technische Universität Braunschweig, 38106 Braunschweig, Germany; a.jukan@tu-bs.de; 3 ATOS Research and Innovation, Next Generation Cloud Lab, 08020 Barcelona, Spain; ana.juan@atos.net; 4 Barcelona Supercomputing Center, Storage System Group, 08034 Barcelona, Spain; anna.queralt@bsc.es; 5 Engineering Sardegna, 09122 Cagliari, Italy; antonio.salis@eng.it; 6 Worldsensing, 08014 Barcelona, Spain; abartoli@worldsensing.com; 7 XLAB d.o.o., SI-1000 Ljubljana, Slovenia; Matija.cankar@xlab.si; 8 SixSq, 1217 Geneva, Switzerland; cristovao.cordeiro@sixsq.com; 9 Sciences and Technology Facilities Council (STFC), Scientific Computing Department, Didcot OX11 0QX, UK; jens.jensen@stfc.ac.uk; 10 Digital Nature, W91 VY70 Clane, Ireland; john@digitalnature.ie

**Keywords:** cloud continuum, fog computing, edge computing, fog-to-cloud, internet of things (IoT)

## Abstract

The wide adoption of the recently coined fog and edge computing paradigms alongside conventional cloud computing creates a novel scenario, known as the cloud continuum, where services may benefit from the overall set of resources to optimize their execution. To operate successfully, such a cloud continuum scenario demands for novel management strategies, enabling a coordinated and efficient management of the entire set of resources, from the edge up to the cloud, designed in particular to address key edge characteristics, such as mobility, heterogeneity and volatility. The design of such a management framework poses many research challenges and has already promoted many initiatives worldwide at different levels. In this paper we present the results of one of these experiences driven by an EU H2020 project, focusing on the lessons learnt from a real deployment of the proposed management solution in three different industrial scenarios. We think that such a description may help understand the benefits brought in by a holistic cloud continuum management and also may help other initiatives in their design and development processes.

## 1. Introducing the Concept

Today, there is no computing-intensive service without a reference to cloud computing. What “the Internet” has been to the end-users to run their web browsers and connect to various websites, is now becoming “the Cloud”, where users run services, exchange data, share family pictures, and join social networks. From a practical perspective, cloud computing provides unlimited computing and storage, ubiquity, efficiency, and elasticity, all in all benefiting from an economy of scale model. However, although cloud computing promised to efficiently support computing tasks required by most services, the technology and services evolution end up demanding additional features not properly supported by cloud. The rationale for such an assessment sits on three key pillars: edge systems, new services and data.

Indeed, nowadays we are facing an unstoppable and ever-growing deployment of end systems, running at the edge of the network, including a large set of devices (smartphones, vehicles, smart city objects, wearables, voice assistants, etc.), endowed with many different characteristics, functionalities, needs and imposed constraints. The impact brought in by the deployment of these devices may be easily inferred from reading some key figures and predictions [1]; the number of IoT devices currently connected to the Internet is estimated at 27 billion devices and is expected to grow up to 75 billion by 2025, it is estimated that the average person will own and use at least 15 connected devices by 2030, 600 million people worldwide use voice-activated devices once a week today, and the global connected home market is expected to grow to over $150 billion by 2023. In this context it is deeply alarming that only 48% of companies are able to detect security breaches in their IoT devices [1].

Thus, in this highly technological scenario, it seems quite obvious that new services will arise benefiting from the capabilities introduced by edge devices, enabling both a new range of smart services fueled by strategies based on user monitoring (physically and emotionally), ending up in highly customized services (in terms of users’ interest, geolocalization, spirit and so on), and more aware consumers, willing to share their *contextual* data as long as they get some value in return, knowing that, as of today 78 percent of consumers perceive data privacy as the main barrier to using connected devices in the future [1]. This infrastructure, data and smartness evolution opens the door to consider a new paradigm shifting the current IoT towards an Internet of Behavior (IoB), intended not to monitor people but to digitally link them to actions, such as for example linking a user’s image to a specific activity or identifying specific users behavior to be penalized or rewarded (e.g., driving), although important ethical issues will need to be carefully considered for deployments of such solutions. It is however with no doubt that a successful deployment of this envisioned scenario will strongly depend on the capacity to organize and process the collected data from its raw form up to its distilled information, that becoming a challenging task when dealing with large amount of distributed data sources producing heterogeneous and non-structured data.

The key question to face is where data must be processed since the answer will drive key research opportunities. It is obvious that IoT data are collected at the edge (i.e., close to the user) and the actions to be implemented for the user (service outcome) are also at the edge, so it seems on evidence that the process must be handled close to where the user and data are, which imposes strong limitations on the utilization of cloud services, located far away from the user. It is also worth noticing that keeping the data close to the user with no need to be forwarded up to and down to and from the cloud to be processed, significantly reduces the network load and also minimizes the chances for a security breach. Consequently, new strategies must be sought intended to take advantage of locality. In this arena, fog [2] and edge [3] computing come to the fore. Although both computing paradigms are conceptually strongly connected, some differences may be identified between them. In short, while fog computing sets its capacities on deploying a (or using an existing) cloud-like infrastructure close to the edge (i.e., the fog) where data processing and smart services are executed, edge computing moves these processing needs to the edge devices themselves. In fact, although some preliminary studies start analyzing the impact edge and fog computing may have when compared to cloud computing [4], neither fog nor edge computing have been conceived to replace the cloud. Rather, they all when put together can be seen as a computing continuum environment, combining the entire set of heterogeneous resources from the edge up to the cloud along with the advantages brought in by these technologies and, therefore, providing enhanced options for efficient smart services execution (see [5,6,7] just to cite a few references in the literature addressing this area).

In short, the envisioned computing continuum scenario brings together a large set of different and heterogeneous resources, all of which must be properly managed to optimize the service execution while simultaneously making the most out of the set of available resources. To this end, a coordinated control and management strategy is needed, designed to smartly manage and allocate resources, be they either in the cloud or at the edge, facilitating an efficient and optimized mapping of resources into services.

This paper is intended to highlight the lessons learnt from a real deployment of the mF2C project outcome [8]. In short, the H2020 mF2C project has gathered a mix of industrial and academic partners to design, implement and validate a coordinated management plane for the envisioned computing continuum, extensively demonstrated and validated in three operational real-world industrial scenarios.

The main contributions of this paper are the following:

- To illustrate the lessons learnt while designing and implementing a coordinated management solution for the cloud continuum;
- To present the results obtained when deploying the proposed solution in real-world scenarios;
- To propose the obtained validation results to serve as a benchmark for other similar initiatives.

This paper is structured as follows: Section 2 refers to the recent efforts in the paper areas. Then, Section 3 introduces the main contributions in the cloud continuum field addressed by the mF2C project, including the challenges, and the proposed architectural framework. Section 4 presents the benefits obtained after deploying the proposed architecture into real industrial use cases. Section 5 highlights the lessons learnt after the entire solution development lifecycle, from the architectural design to the implementation, deployment and validation analysis. Finally, Section 6 concludes the paper. Finally, it must be also highlighted that all materials and methods described in this paper may be found and downloaded from the mF2C project website [8].

## 2. Review of the State-of-the-Art

This section revisits recent literature in fields closely linked to the proposed mF2C architecture. Nowadays, the envisioned effects of the cloud continuum in a wide range of scenarios, from smart environment scenarios to future 5G scenarios, have fueled several initiatives aimed at resolving the different challenges posed by their potential deployment, including the Open Fog Consortium [9] (now in the Industrial Internet Consortium), ETSI Multi-access Edge Computing (MEC) [10], as well as significant research efforts, including Fog-to-Cloud computing (F2C) [11], resources continuum [12], serverless computing [13], osmotic computing [14] and cloud-to-thing continuum [15], in the recent literature. Aligned to this technological push, some European research projects funded within the H2020 program have been successfully executed addressing related research areas, such as INTER-IoT [16] (aimed at integrating heterogeneous IoT platforms), RECAP [17] (focusing on automating distributed cloud infrastructure management), mF2C [8] (proposing a solution for the cloud continuum management), DITAS [18], (focusing on data intensive applications and exploring coordinated data and computation movement strategies), lightkone [19] (developing a general-purpose computation model for edge networks), CLASS [20] (intended to coordinating edge and cloud for big data analytics) or DRUID-NET [21] (proposing novel resource allocation mechanisms for edge systems).

At the same time, many contributions in the recent literature address well-known research challenges in the cloud continuum area, including: (i) FogOS [22], the OpenFog Reference Architecture (OFRA) [23], Foglets [24], Edge-as-a-Service (EaaS) [25], as well as the contributions in [26,27,28], all dealing with resource management; (ii) the architecture for mobile crowdsensing [29] as well as Azure Stack and Azure IoT Edge Edge [30], addressing aspects related to traffic offloading; (iii) Ibis [31], Swift [32], Taverna [33], Pegasus [34] or COMPSs [35], as works related to programming models; iv) CloudPath [36], FogStore [37], DataFog [38], eXtremeDB [39], IBM Informix [40], Redis Enterprise [41] and HarperDB [42] as well as the open-source YottaDB [43] and dataClay [44], for data management, and; (v) the works in [45,46,47,48,49,50,51] particularly proposing strategies for resources allocation.

Finally, it is worth mentioning that the progress from commercial actors is not negligible. Major Cloud vendors such as Amazon Web Services and Azure are taking the Edge computing opportunity to extend their cloud services and to provide an additional entry point for their consumption. Azure IoT Edge [52] is available as Open Source platform [53]. AWS Greengrass [54] software stack is available for both ARM and x86 devices with minimum required capacity.

## 3. mF2C: Managing the Cloud Continuum

This section summarizes the work done towards a cloud continuum management framework within the mF2C project, designed to properly manage the huge, heterogeneous, volatile and dynamic set of resources from the edge up to the cloud, and intended to optimize services execution. To this end, key technical challenges are first identified before introducing the architectural definition –a more detailed description may be found in [8].

### 3.1. Key Technical Challenges

A well-defined set of technical challenges must be addressed for a successful development of the proposed mF2C management framework:

- The proposed solution must leverage a distributed and decentralized architecture, intended to overcome the limitations of a centralized cloud-based approach in terms of efficiency, agility and security;
- A coordinated orchestration is required to generate individual service workflows, to map these service workflows into those resources best suited for each of the requested services, and to coordinate the interactions among the different devices involved in the service execution. This coordination must be transparent to the user;
- The proposed management framework must enable a timely creation of dynamically provisioned cloud continuum infrastructure, automatic discovery of resources, context-based decision-making, stateless communication and transparent connectivity;
- The dynamic provisioning also requires advanced service scheduling to decide how the service’s individual functions are split across different devices and mapped into different physical resources. Dynamic refinements of the scheduling should be possible based on runtime conditions;
- The envisioned dynamic mapping strategy requires a high-performance service execution through parallel computing to accommodate resource volatility and real-time service demands;
- An appropriate runtime system for the envisioned novel service execution paradigm shall be considered to optimize the characteristics of the resources brought by the combination of fog and cloud computing;
- Security and privacy need to address critical security requirements for coordinating and managing distributed components within the cloud continuum scenario;
- The proposed architectural framework must be able to address the mobility and scalability implications of service execution, interaction and communication among the different devices;

### 3.2. Proposed Architecture

**Figure 1:**
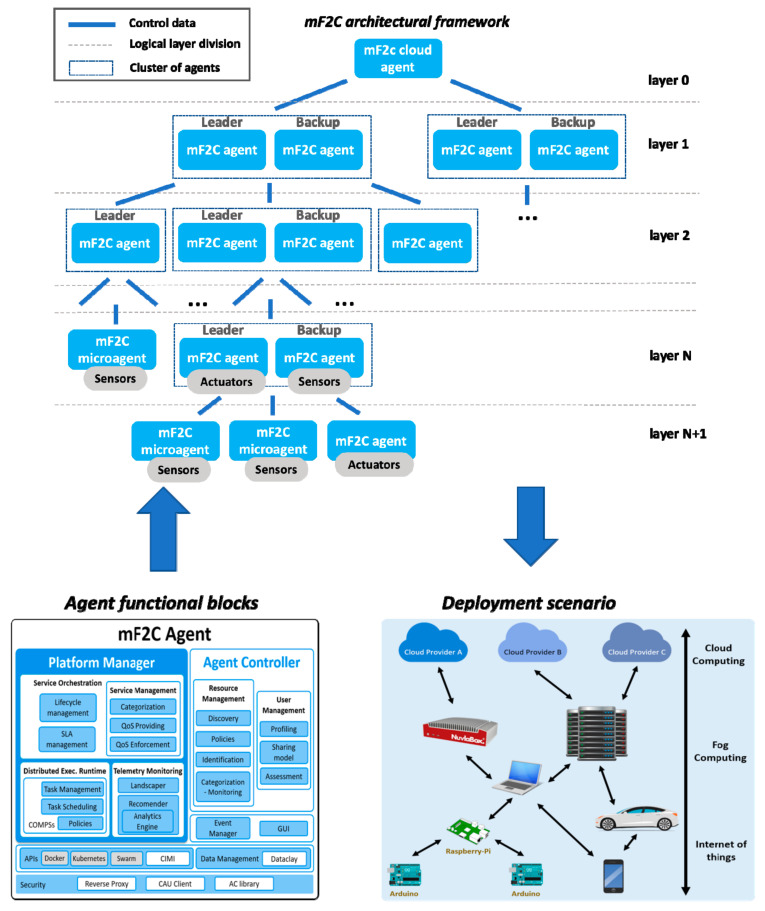
mF2C layered architecture, agents blocks and envisioned cloud continuum scenario.

The mF2C solution proposes a coordinated management strategy capable of leveraging all existing and potentially available resources in the cloud continuum, from the edge up to the cloud, to execute a service. To this end, the mF2C system proposes a layered and hierarchical architecture (Figure 1) where resources are categorized, using a so-called agent entity to deploy the management functionalities in every mF2C component. The architecture is divided into different logical layers: from layer 0, at the cloud, to layer N+1, the closest to the edge, where three different kinds of software entities are deployed: agent, cloud agent and microagent. In a nutshell, the agent is the one used by default in most of the devices of the architecture, the cloud agent is a slightly modified version of the standard agent adapted for the cloud that can be instantiated over one or multiple private or public clouds according to the specific requirements of the system, and the microagent is a simplified version of the agent designed to be used by highly constrained devices with no capacity to support a fully operative agent.

Below layer 0 (cloud), the instantiation of multiple agents will start with the creation of a layered mF2C architecture, enabling different agents to be grouped turning into multiple clusters, having at least one leader (cluster head) and if possible one backup for resilience purposes (see Figure 1). In the last layer of every branch we find either agents with no devices attached or microagents deployed in highly constrained devices. Indeed, microagents can be placed in any layer in the architecture, but without the possibility of managing other agents, acting as a leaf in a tree hierarchy.

When an agent receives a request for executing a service (regardless of the layer), the agent proceeds as follows: if the requested agent has the required resources itself, the service will be executed in that agent; otherwise, the service request will be forwarded to the leader in the layer above. If the service execution arrives at an agent which controls multiple other agents within lower layers, the agent will first try to allocate the service using those resources, and if impossible, it will forward the request to the upper layer in the hierarchy.

Agents execute services from a services port-folio reachable by the user logged in through the mF2C dashboard (GUI). The proposed management solution must guarantee that services are executed meeting the required Quality of Service (QoS) as identified within the Service Level Agreement (SLA) between the user and the provider. To this end, and in order to maximize the guarantees for the SLA to be met, QoS functionalities are split into two different components: (i) the QoS providing, responsible for both defining the resources conditions to meet specific QoS requirements and reporting on past SLA violations and; (ii) the QoS enforcing responsible for deploying actions intended to meet QoS at runtime, e.g., reconfiguring resources, tasks, services, etc., in real-time while the service is being executed. Thus, an AI-assisted estimator is defined to predict what the delivered QoS will be in runtime.

Figure 1 also depicts the functional blocks defined for the agent entity, Platform Manager (PM), Agent Controller (AC), Data Management, Security, Event Manager, Graphical User Interface (GUI) and an Application Programming Interface (API) as an entry point. From an implementation perspective an agent is deployed as a collection of Docker containers, with each block exposed via a single REST interface based on CIMI [55]. These blocks are briefly described next.

The Platform Manager component is a global entity acting as a controller for agents in lower layers, and a receiver of control data, when it is being managed by agents from upper layers. It is responsible for service orchestration, telemetry data monitoring from different sources and the coordination of the end-user applications execution. The Agent Controller encompasses all functionalities dealing with the resource and user management of local resources, being responsible for defining and executing the assessment of the user’s device profile and its sharing model. The role of the Data Management focuses on organizing all mF2C system data resources and making them accessible for the appropriate devices. The Event Manager is an event tracking module representing a broker that will be used by each of the modules to publish/subscribe to events, e.g., service deployed, device added/removed, etc. Security is provided through three different components: trust (using a Control Area Unit, CAU [56]), web application endpoint security (using inherited CIMI standard security mechanisms along with a reverse proxy) and data protection (using a Security Library providing methods for creating message token based on the security level required). The GUI will facilitate users interaction with the platform (registration and management) and services operation (registration, catalogue, access, launch). The API provided by CIMI represents the main entry point for any mF2C component.

### 3.3. Addressing the Challenges

Based on the challenges and requirements previously identified in this section, Table 1 highlights the different strategies mF2C utilizes and deploys to take over each one of them, linking them all to the responsible mF2C architectural component or functional block.

## 4. Performance Results

The validation strategy starts by identifying three cloud continuum scenarios where a suitable cloud continuum management solution may undoubtedly bring many benefits in. The validation process encompasses both real deployment and emulated scenarios accurately replicating the real world when an operational deployment is not possible. This section elaborates on the scenario descriptions, highlighting the benefits in terms of well-defined KPIs obtained after a successful deployment of the mF2C solution. It is worth mentioning that the evaluation outcome presented in this paper is just a short summary of the real validation activity carried out to validate the mF2C solution. Readers willing to know more about the validation results may visit the different project deliverables publicly available at the project website [8].

### 4.1. Scenario 1: Emergency Situation Management in Smart Cities (ESM)

**Figure 2:**
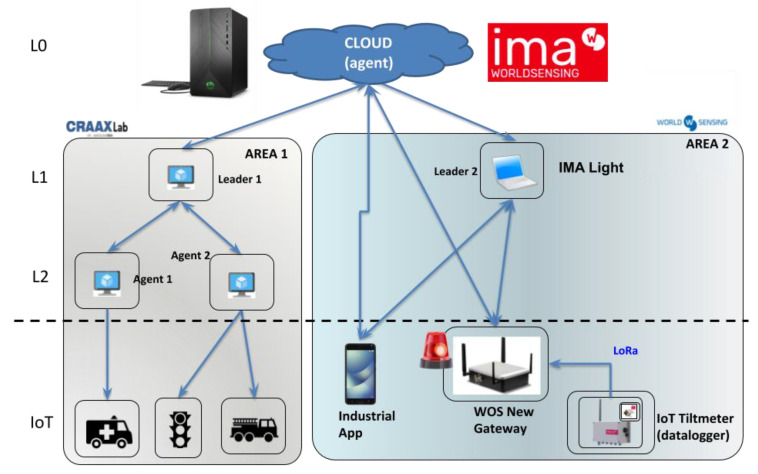
mF2C deployed at the ESM use case.

This first scenario explores the use of the mF2C management framework in the context of smart/connected IoT and Industry 4.0 applications in a smart city. Particularly, the scenario considers an Emergency Situation Management (ESM) application, particularly applied to the monitoring of infrastructures construction. In a nutshell, the application analyses the flow of assets within smart infrastructures (i.e., enriched with IoT devices), in order to: (i) provide useful information to infrastructure operators; (ii) detect potential emergency situations in a real-time basis, and; (iii) decrease the necessary resources in terms of energy, latency, etc. to efficiently respond to these situations in accordance with the application’s requirements. Figure 2 depicts the proposed validation scenario, including the deployment of the mF2C architectural components into the emulated topology. Table 2 shows the obtained results in terms of quantifiable KPIs. From a deployment perspective, Figure 2 shows the layered architecture and the two areas the validation scenario is split into. While the area on the left includes the actuation systems, the area on the right refers to where the emergency occurs and includes the sensing systems, both areas being connected through the cloud (layer 0) where the ESM application (so-called Industrial Monitoring Application, IMA) is running.

The storyline of this validation scenario is as follows: the location of construction workers is periodically forwarded to IMA (cloud) (GDPR compliance is granted through requiring consent forms, using only tracking mobile devices provided by the company, not displaying the location except in case of an emergency and not associating a specific worker to a specific device –since the only requirement is locating the device closest to the emergency). The tiltmeter sensor located in the infrastructure (edge) to be monitored, regularly sends data to the Monitoring Software. Once the Monitoring Software detects that a tiltmeter threshold is exceeded (i.e., the infrastructure collapses), the response plan is activated (moving to area 1) acting as: (i) first the sirens are started; (ii) then, the closest worker (identified through the location information stored previously) is asked to go and confirm or cancel the emergency using the mobile device; (iii) the authorities and the relevant actors are then alerted, and the alerts visualized on the dashboard and maps in real time; (iv) the warnings are emitted, and the emergency vehicles are sent, through the optimum path calculated at that moment, and finally; (v) the traffic lights are changed to green on the path of the emergency vehicles in order to optimize the intervention time.

Knowing the fact that this scenario plays with data and devices located within the cloud continuum and also demands for immediate reaction, the proposed mF2C solution is expected to show clear benefits, as shown in the KPIs evaluation in Table 2, where measured results for latency, reliability/QoS and CAPEX savings are quantified.

### 4.2. Scenario 2: Smart Boat Service (SBS)

This scenario falls into the management of IoT devices and sensors intended to provide safer navigation in the marine sector. The example demonstrated not just making all the ship’s sensors work together, processing and correlating the collected data in a combined cloud continuum system, but also interacting with external data sources such as other ships and marine vehicles and satellites.

**Figure 3:**
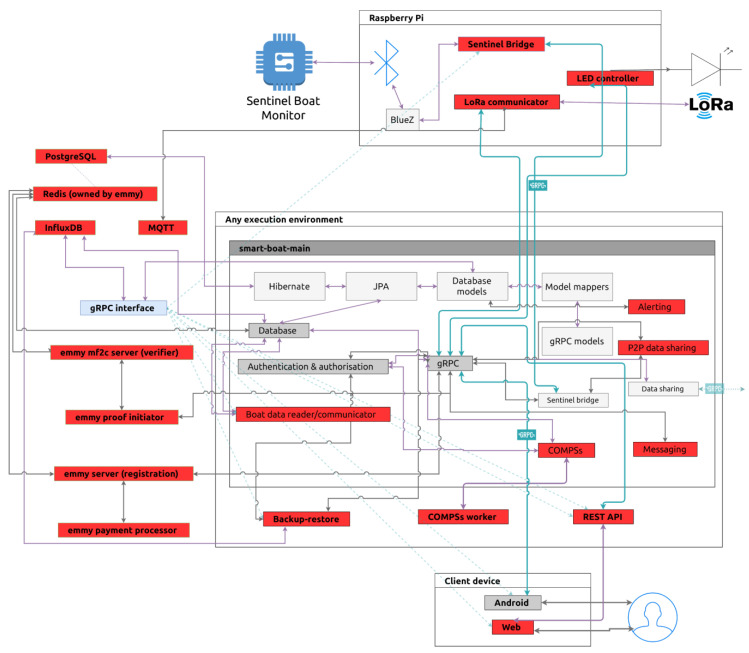
mF2C deployed at the SBS use case: Simplified diagram of the SBS architecture.

To have a vivid sense of the target users and their interaction with the Smart Boat software (the application users are endowed with to run this scenario), five potential storylines are considered: continuous boat monitoring, LoRa distress call, online docking and anchoring reservation, and remote light management (more details may be found in [8]). Figure 3 includes a description of the mF2C solution deployment on the SBS scenario, including the different technologies and systems inherent to the SBS that are considered (e.g., Sentinel Boat Monitor, Sentinel Hub). The different functionalities to be evaluated in this scenario, inferred from the storylines defined above, are represented in terms of workflow diagrams (see [8] for a detailed description). To validate the mF2C system in the SBS scenario, several tests were carried out in various setups to verify and validate whether the mF2C system is fully capable of handling the cloud continuum scenario and quantify its benefits for this particular application. These tests were performed both in a lab environment and on-location, on a yacht, in order to have both the necessary flexibility in deployment and the ability to validate the technology on actual equipment, facing real-world challenges. Table 3 shows the benefits brought in by deploying mF2C to manage the cloud continuum in this particular scenario, through quantifiable KPIs.

### 4.3. Scenario 3: Smart Fog-Hub Service (SFHS)

**Figure 4:**
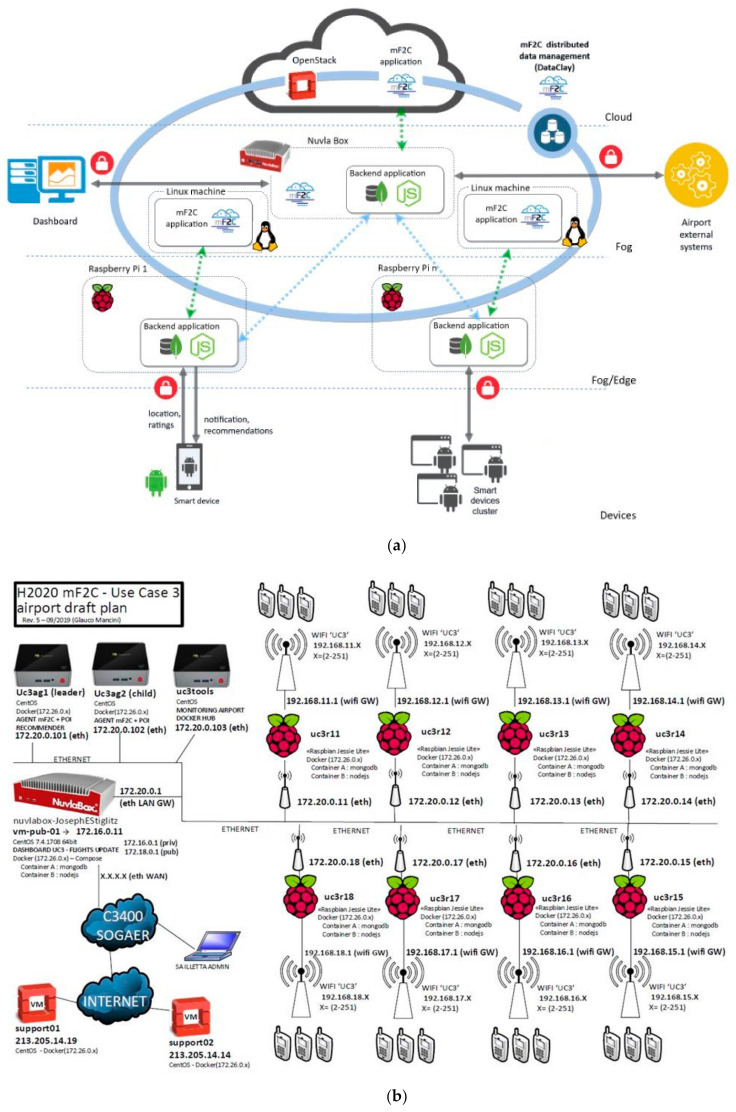
mF2C deployed at the SBS use case: (**a**) SFHS architecture; (**b**) Final systems diagram.

The third scenario extends the concept of a “cloud hub” to a novel “fog hub”, driven by real market needs. The proposed setup assumes that value is generated at the business services level, particularly in spaces with recurring concentrations of people and objects that can communicate and interact with each other (e.g., airports, railway stations, seaports, shopping centers, hospitals, sports facilities, large parking areas, but also domestic scenarios with a communal clustering level). The scenario proposes to set up (fog) hubs to interact with all objects within the area of coverage, and to operate “in-proximity” marketing efforts, applying predictive algorithms to track (in an anonymized form) movements, choices and decisions of persons nearby, or even extend the hub with devices (e.g., beacons) capable of sending inputs (e.g., customized advertising) and determine the effectiveness of the specific campaign in terms of attention/visits (purchasing products or services). To build this scenario a real deployment of hardware (Raspberry Pi) and software (mF2C and a user Android app developed in the project) has been deployed at the Cagliari Airport in Sardinia. Figure 4 shows a detailed view of the SFHS deployment, including the final SFHS logical architecture with the different architectural components and visualizing the different layers (Figure 4a), and the physical deployment with the real systems (Figure 4b).

From a user oriented perspective, the main objective in this scenario is to endow the end user with a friendly and interactive experience during the stay and moving in the airport field, using the Android app specifically designed and developed for this purpose. This aim is materialized in the following functionalities: (i) proximity computation of Points of Interest (POI) and notification delivery to the user; (ii) subscription for notifications on the traveler flight; (iii) subscription for notifications on specific topics of interest to the user; (iv) rating tool for the POI (through the app); (v) topics advisor fed by historical end user ratings, and; (vii) administrative dashboard for system and user behavior monitoring. This user oriented objective is mapped into two main tasks to be deployed as mF2C services, proximity computation and recommendation of POI.

From a global perspective, the adoption of the mF2C system to handle the combined set of resources in the cloud continuum, brings two main benefits. First, it is possible to handle both up and down scaling, according to the number of simultaneous users to manage. Second, the mF2C system is able to manage several fog areas in more complex scenarios like smart cities (e.g., a fog area in the airport, another in the train station, another in specific tourist places of interest in the city, etc.), hence enlarging the services portfolio to include much more complex services.

The tests carried out to validate the mF2C solution in this scenario are designed to clearly visualizing in a quantified manner the benefits brought in by an mF2C deployment to manage the cloud continuum. To this end, response time, resilience and data locality (GDPR) are analyzed, as shown in Table 4.

## 5. Lessons Learnt from a Real Deployment

As detailed in the previous section, the whole integrated mF2C management framework has been deployed in three real-world scenarios to demonstrate the benefits such a solution may bring to smartly and efficiently manage the whole set of cloud continuum resources. Indeed, integrating the entire set of blocks, components and modules to guarantee a single mF2C version offering all expected functionalities was a challenging task, with valuable lessons learnt. In fact, in this section we dig into these lessons learnt with the aim that this exercise can be useful for similar initiatives in the field.

Two main key areas, referred to as focus areas in the following, may be conceived when reviewing the work done. The first area is related to the mF2C design and implementation, and the second one to the deployment in the use cases and the end users experience.

### 5.1. Focus Area on Design and Implementation

The mF2C concept was designed to be a decentralized and hierarchical architecture to be deployed on a highly heterogeneous and volatile scenario. Actually, the fog definition, as stated in [6] already identifies fog as hierarchical. This approach, although seemingly obvious, fueled large discussions on its benefits. Thus, being aware of its benefits and limitations, a flat-based architecture may be designed instead, where clusters and areas may be dynamically set and reconfigured to rapidly and nimbly react to the quantity and volatility of devices. However, the trade-off between scalability and efficiency should be analyzed and compared for both architectural proposals to end up with the best suited architecture.

Certainly, although the hierarchical approach was assumed for scalability purposes, some restrictions still apply, for example for horizontal communication and data accuracy, so a very clear policy must be sought to identify clusters and hierarchical levels to reduce overhead while facilitating communication, interoperability and accuracy. In fact, the loss of accuracy motivated by the mandatory data aggregation must be minimized to avoid errors, for example in the topology available to run a service. It is also important to define a coherent and consistent resources and services categorization mechanism, so the mapping of services into resources may perfectly suit what the needs and capacities are respectively. That said, it is not even obvious that a single approach may suit all potential infrastructure and services scenarios. Areas mixing both approaches (flat and hierarchical), may be also thought to handle different needs and requirements, being aware of the overhead added to facilitate interoperability.

The mF2C project decided to use CIMI as the main system interface assuming a modular deployment based on docker containers. There are still some doubts about CIMI optimality for this particular scenario, which is not only about cloud (the one CIMI was designed for) but also fog and edge, mainly fueled by the resources needed and for its performance in real-time setups.

From a more device oriented perspective, efforts in the area must consider discovery as a key pillar and go far beyond Wi-Fi based solutions to detect devices behavior, to extend the platform scope. Mobility patterns and prediction strategies must be added to optimize resources discovery and reduce volatility. In fact, once the project is over, we agree that predictive (AI-assisted) models should be added, intended to estimate devices motion and capacity and even training models based on previously collected data and/or patterns. These predictive models may also be considered to generate QoS estimation models and predict how quality would evolve for specific users and services, thus facilitating a more accurate mapping and re-configuration of resources into services.

Finally, it is a must to consider security and privacy from the early design phases. Actually, implementing security by design takes more time and effort than just implementing the basic application functionality. Worse, projects are often tempted to prioritize basic functionalities over security, not because they do not want to secure it, but because consuming time and effort implementing and improving functionality offer tangible benefits, as functionality can be easily demonstrated. Privacy by design also requires software architects and developers to take privacy in general and GDPR in particular into consideration in their designs and code to ensure that sensitive data are handled appropriate and in accordance with GDPR directives.

### 5.2. Focus Area on Deployment and End User Experience

The first lesson here refers to the integration process. Indeed, being such a large system encompassing many different functionalities and versions, a smooth and well-defined integration process must be defined, aimed not only at facilitating the integration of different components but also at easing the refinement of iterative versions. Assuming the large diversity of devices building the cloud continuum scenario, different versions of the main mF2C delivery artifact (the Agent) must be designed and implemented. To this end, the agent was deployed first including all defined functionalities and then a light version was designed, the microagent, to be deployed in highly constrained devices, those not having sufficient resources to run the full agent, and thus including only the minimum set of functionalities for mF2C to operate. In fact, this need adds burden into the overall deployment process, since the interoperation among the different suites must be appropriate to enable all offered functionalities to be executed. Indeed, a policy must be agreed to identify the minimum set of capacities a resource must have to become mF2C friendly, guaranteeing that the resulting deployment may be supported by very basic devices. Either way, it is imperative to follow a modular approach for the agent code design, enabling an easy deployment of the different agent suites.

Finally, it is also worth noticing that the configuration of the mF2C platform must be straight-forward to avoid users refusing its adoption, while maintain guarantees on performance, security and privacy.

## 6. Conclusions and Opportunities

This paper presents results obtained from deploying a real management framework for the cloud continuum, emphasizing lessons learnt during the whole system deployment lifecycle in three different use cases. The main conclusion obtained after this join effort boil down to highlighting both the complexity to build a widely adopted platform that can support a large set of scenarios and applications, and the fact that some challenges remain unsolved, but which may significantly benefit from adoption of predictive, AI-assisted strategies. The validation scenario, built upon three real-world industrial cases, is shown not only to validate the proposed management framework but also functions as a form of benchmark for other initiatives to help them on their validation processes.

Being a challenging and continuously evolving environment, many additional research opportunities may yet arise. Clearly, AI-assisted strategies might be efficiently applied to optimize many of the processes included in the proposed architecture (devices discovery, QoS management, topology mapping, optimal runtime, etc.). Moreover, it is also worth mentioning that the features introduced by a management framework like mF2C, enable the deployment of novel collaborative models, supported by, for example, disruptive sharing policies not only at user but also at industrial level. These can optimize execution of existing and future services and fuel new business models into the future.

Finally, future work is driving on three main directions. First, there is plenty of space for contributions intended to improve strategies to be deployed on any functional block in the proposed architecture. For example, new prediction schemes may be included to estimate mobile devices motion, hence generating patterns that may be used for policies deployed for devices clustering or even for pre-fetching resources. Second, additional efforts must be allocated to improve security guarantees. Although some preliminary work has been done in the project, novel AI-assisted solutions must be considered to make the system cyber resilient, incorporating self-management solutions for detection, self-healing and self-recovery. The last but not the least effort should go to extend the proposed management solution with additional functionalities, making this management architecture to become a fully meta-OS, facilitating both the overall management of resources and services, from the perspective of software developers, infrastructure providers and users, and the development of innovative resources sharing models.

## Figures and Tables

**Table 1:** mF2C strategy to face defined challenges and requirements.

Challenge	Technical Requirement	mF2C Strategy
Coordinated orchestration	Individual service workflows	The Task Management detects application’ tasks, finds data dependencies between them and builds a task graph or workflow
	Efficient mapping of workflows into resources	Services and resources are categorized in the Platform Manager and the Agent Controller modules respectively, and the Recommender issues the proper set of resources to execute a service. Then, the Task Management and Scheduling blocks allocate the mapping also according to the feedback from the QoS and Lifecycle components
	Coordinated operation of all devices involved in service execution	The Lifecycle components in the Platform Manager handle the actions required to enable a coordinated operation of the whole set of actions
	User agnostic operation	Services are orchestrated with no need for the user to intervene. In fact, the user should only identify devices used to connect, profile and sharing model (if any)
	Volatility	The Recommender and the Discovery process handle the unavoidable volatility caused by systems mobility
Infrastructure adaptability	Dynamic infrastructure provisioning	Clusters of devices may be dynamically reconfigured (reduced/extended) to face devices mobility, through the proposed resources discovery strategy. Resources can also be re-provisioned to meet QoS enforcing.
	Automatic resources discovery	Devices serving as leaders use their Wi-Fi interface to broadcast beacons containing mF2C-specific Vendor-Specific Information Elements (VSIEs) and regular agents perform a Wi-Fi scan looking for beacons containing mF2C VSIEs to detect the presence of nearby leaders.
	Context based decision making	The Landscaper component contains an updated and accurate view of the physical and logical topology, used by the Recommender to decide on the resources best suited to execute a particular service.
	Transparent connectivity	The proposed hierarchical approach, setting clusters and branches with a clear horizontal and vertical communication strategy, facilitates a completely transparent connectivity.
	Adequate services scheduling	The Task Scheduling schedules the different tasks in the distributed computing platform, leveraging the Recommender outcome, where the required data is and which resources can perform the specific task.
	High performance service execution	A parallel computing approach, based on COMPSs [35] is considered in the Task Scheduling component to facilitate the simultaneous execution of different tasks in non-concurrent workflows
Appropriate Runtime system	Resources characteristics optimization	The Landscaper and the Recommender components are responsible for keeping an accurate view of the available resources and to “recommend” what resources will best suit specific service needs, respectively.
	QoS guarantees	QoS providing and QoS enforcing blocks are responsible for identifying the resources needed to meet the expected QoS and for adapting that set of resources to real-time needs in runtime respectively. SLA policies are also proposed to meet specific mF2C and cloud continuum requirements.
Security and privacy provisioning	Security guarantees	mF2C follows a secure by design approach, consisting in using: (i) appropriate tools for security development (e.g., DAST); (ii) DevSecOps; (iii) design strategies based on well-maintained libraries (e.g., OpenSSL), standard interfaces (e.g., GSSAPI) and standard protocols/formats (e.g., HTTPS, OAuth2, OIDC, JWT); (iv) securing fog-to-fog, inter-fog and fog-to-cloud communications via encrypting messages (e.g., AES, RSA) and well-designed APIs (e.g., CIMI).
	Privacy guarantees	Confidentiality and integrity are protected in intra-agent communications through the default private Docker network. Encryption and secure transport protocols are used for external agent communications. GDPR compliance for user data is handled through a consent process supported by message token generation, cryptography and access control.
Scalability, viability and resilience	Mobility impact on services execution	Mobility impacts on QoS, and the QoS enforcement component is responsible for predicting the need for additional resources in advance, which are dynamically added to the execution by the Policies block.
	Devices communication and interaction	CIMI is used to facilitate mF2C systems communication and interaction, either through the invocation of functionalities in other components or through the access to shared data.
	Scalable solution	Dataclay [44] as a distributed solution to handle huge data volumes, and COMPSs [35] as a distributed execution runtime supporting the Task Management and Scheduling components, are used for scalability purposes.
	Highly constrained devices	The microagent version of the agent is specifically designed to be deployed on highly constrained devices.
	Resilient solution	The mF2C solution includes a set of policies to handle devices failures that may interrupt global performance, mainly focusing on leader nodes.

**Table 2:** KPIs summary for the ESM use case.

KPI	Description	Result
Latency	Measurement of the time it takes for the physical alarm to be started when a measurement exceeds a threshold	The mF2C environment provides an improvement of about 76% compared to the cloud only environment
Reliability/QoS	The end user application is based on continuous data communication, intrinsic redundancy of the mF2C architecture, guarantees better use of bandwidth and resilience capabilities	Availability increased to 90% with the use of the mF2C environment. This represents a 10% improvement of the commercial offer.
CAPEX savings	Economic impact of the mF2C system compared to an alternative architecture where a cloud-based application is enriched with a powerful Fog device at the edge level	This represents CAPEX savings of at least 17%

**Table 3:** KPIs summary for the SBS use case.

KPI/Target	Description	Result
Expanded Coverage/20%	Expanded coverage of transmitting Smart Boat sensor data to cloud by using fog devices and LoRa communication.	Successfully achieved with a multi-hop solution
Improved Stability/20%	Facilitate usage of fog devices and boat to boat communication enables functionalities in dark zones without coverage.	Boat can be monitored successfully using LoRa also without 3G/4G network over large period of time (days, weeks).
More Responsive Applications	Communication response time improvement using mF2C instead of cloud	Mobile device—mF2C latency is 2.5x lower than mobile device—cloud.
Lower Operational Cost and Potential Improvement of ROI	Usage of mF2C in software development provides some tools in the package. This means a lower number of tools to maintain for development and running the application	As mF2C brings its own cloud and fog deployment platform, its own orchestration engine on agents and an abstracted way of sensor interaction, we estimated that 15–20% fewer resources are required for development and operation.
Contribution to Safety on Sea	Safety on sea is a prime concern of our customers. With an expanded coverage and improved stability of the application this KPI can be achieved.	The application provides communication between the boats in no network coverage zones and allows transmitting sensor data from no coverage zones to the cloud. The KPI is largely achieved.

**Table 4:** KPIs summary for the SFHS use case.

KPI	Description	Result
Response Time	The end user application requires real-time response, end-to-end response time for the proximity calculation is performed balancing fog and cloud processing to obtain better response times.	The mF2C environment provides an improvement of at least 15% compared to the cloud only environment.
Resilience	The end user application is based on continuous data communication, intrinsic redundancy of the mF2C architecture, guarantees better use of bandwidth and resilience capabilities.	At least 3RPI connect to each device with handover capability to link to the strongest signal in the field, so mF2C guarantees better coverage, resilience capabilities and better use of bandwidth.
Data Locality and Regulatory Compliance	Processing of personal data is done at the edge using security/privacy by design mF2C features, thus fulfilling the GDPR constraints (compliant or not).	Full GDPR compliancy and control on personal data management.

## Data Availability

Not applicable.

## Abbreviations

The next abbreviations are used in this paper.

AC	Agent Controller
AI	Artificial Intelligence
API	Application Programming Interface
AWS	Amazon Web Services
CAPEX	Capital Expenditure
CAU	Control Area unit
CIMI	Cloud Infrastructure Management Interface
ESM	Emergency Situation Management
ETSI	European Telecommunications Standard Institute
F2C	Fog-to-Cloud
GDPR	General Data Protection Regulation
GUI	Graphical User Interface
IMA	Industrial Monitoring Application
IoB	Internet of Behavior
IoT	Internet of Things
KPI	Key Performance Indicators
LoRa	Long Range
MEC	Multi-Access Edge Computing
OFRA	OpenFog Reference Architecture
POI	Point of Interest
PM	Platform Manager
QoS	Quality of Service
RPI	Raspberry Pi
SBS	Smart Boat Service
SFHS	Smart Fog-Hub Service
SLA	Service Level Agreement
